# Vaccination with pre-diagnostic tumor antigens but not established tumor antigens inhibits spontaneous tumorigenesis in TgMMTV-neu mice

**DOI:** 10.1186/2051-1426-1-S1-P237

**Published:** 2013-11-07

**Authors:** Sasha E Stanton, Jianning Mao, John Ladd, Lauren Rastedder, Ekram Gad, Samir Hanash, Hailing Lu, Mary L Disis

**Affiliations:** 1 Oncology, University of Washington, Seattle, WA, USA; 2 Fred Hutchinson Cancer Research Center, Seattle, WA, USA; 3 MD Anderson Cancer Center, Houston, TX, USA

## 

Breast cancer is immunogenic and proteins expressed in breast cancer stimulate both an antibody and a T-cell immune response. Cancer related proteins that are aberrant enough to trigger early immune recognition might be ideal targets for a vaccine aimed at preventing the development of breast cancer. Antigen discovery has focused on the identification of immunogenic proteins in tumor bearing individuals; in this study we have used SEREX and a transgenic mouse model to identify immunogenic proteins in animals prior to the development of cancer. These antigens may represent the earliest alterations of the malignant transformation. We identified 6 antigens that were present in mice prior to the development of mammary cancer (Pdhx, Otud6b, Stk39, Zpf238, Lgals8, and Vps35). These proteins were associated with inflammation, autoimmunity, and cellular homeostasis, were highly homologous to human, and were overexpressed in ductal carcinoma in situ and invasive breast cancer in women. We questioned whether these antigens could mediate anti-tumor immunity. When TgMMTV-neu mice (n=5/group) were vaccinated with the individual antigens, vaccination with Pdhx inhibited tumor growth by 62.1%, Otud6B inhibited tumor growth by 23.5%, and Stk39 inhibited tumor growth by 50.3% as compared to empty vector vaccinated control at 27 weeks (p<0.001 for each individual antigen as compared to empty vector). Spontaneous tumorigenesis was inhibited in TgMMTV-neu mice (n=20 mice/group) vaccinated with a panel of three of the early tumor antigens (Pdhx, Otud6b, and Stk39). This panel of antigens inhibited tumor growth by 34.8% by 37 weeks as compared to vector vaccinated mice (p=0.02). Tumorigenesis was similar to empty vector when animals were vaccinated with a panel of antigens that were discovered by using sera derived from animals with established tumors previously identified in our laboratory (Arhgef2, Swap70, and GSN). At 37 weeks the mice vaccinated with the established tumor vaccine had similar growth as compared to vector-vaccinated mice (p=0.69) but 31.1% more growth than the early tumor antigen vaccinated mice (p=0.005). These data suggest vaccination against a panel of pre-invasive breast tumor proteins inhibit tumorigenesis in mice unlike vaccination with a panel of established tumor proteins, suggesting that these pre-invasive antigens are better targets for a preventative vaccine. Future studies will address if these antigens elicit T-cell responses in patients with high-risk breast lesions.

